# Study protocol: a mixed methods feasibility study for a loaded self-managed exercise programme for patellofemoral pain

**DOI:** 10.1186/s40814-017-0167-2

**Published:** 2017-07-20

**Authors:** Benjamin E. Smith, Paul Hendrick, Marcus Bateman, Fiona Moffatt, Michael Skovdal Rathleff, James Selfe, Toby O. Smith, Pip Logan

**Affiliations:** 1 Derby Teaching Hospitals NHS Foundation Trust, Physiotherapy Department (Level 3), London Road Community Hospital, Derby, DE1 2QY UK; 2 Division of Rehabilitation and Ageing, School of Medicine, University of Nottingham, Nottingham, UK; 3 Division of Physiotherapy and Rehabilitation Sciences, School of Health Sciences, University of Nottingham, Nottingham University Hospitals (City Campus), Nottingham, UK; 4 Research Unit for General Practice in Aalborg, Department of Clinical Medicine at Aalborg University, Aalborg, Denmark; 5 Department of Occupational Therapy and Physiotherapy, Department of Clinical Medicine, Aalborg University Hospital, Aalborg, Denmark; 6 Department of Health Professions, Manchester Metropolitan University, Manchester, UK; 7 Faculty of Medicine and Health Sciences, University of East Anglia, Norwich, UK

**Keywords:** Mixed-methods study, Feasibility, Patellofemoral pain, Anterior knee pain, Exercise therapy

## Abstract

**Background:**

Patellofemoral pain (PFP) is one of the most common forms of knee pain in adults under the age of 40, with a prevalence of 23% in the general population. The long-term prognosis is poor, with only one third of people pain-free 1 year after diagnosis.

The biomedical model of pain in relation to persistent PFP has recently been called into question. It has been suggested that interventions for chronic musculoskeletal conditions should consider alternative mechanisms of action, beyond muscles and joints. Modern treatment therapies should consider desensitising strategies, with exercises that target movements and activities patients find fearful and painful.

High-quality research on exercise prescription in relation to pain mechanisms, not directed at specific tissue pathology, and dose response clearly warrants further investigation.

Our primary aim is to establish the feasibility and acceptability of conducting a definitive RCT which will evaluate the clinical and cost-effectiveness of a loaded self-managed exercise programme for people with patellofemoral pain.

**Method:**

This is a single-centred, multiphase, sequential, mixed-methods trial that will evaluate the feasibility of running a definitive large-scale randomised controlled trial of a loaded self-managed exercise programme versus usual physiotherapy. Initially, 8–10 participants with a minimum 3-month history of PFP will be recruited from an NHS physiotherapy waiting list and interviewed. Participants will be invited to discuss perceived barriers and facilitators to exercise engagement, and the meaning and impact of PFP. Then, 60 participants will be recruited in the same manner for the main phase of the feasibility trial. A web-based service will randomise patients to a loaded self-managed exercise programme or usual physiotherapy. The loaded self-managed exercise programme is aimed at addressing lower limb knee and hip weakness and is positioned within a framework of reducing fear/avoidance with an emphasis on self-management. Baseline assessment will include demographic data, average pain within the last week (VAS), fear avoidance behaviours, catastrophising, self-efficacy, sport and leisure activity participation, and general quality of life. Follow-up will be 3 and 6 months. The analysis will focus on descriptive statistics and confidence intervals. The qualitative components will follow a thematic analysis approach.

**Discussion:**

This study will evaluate the feasibility of running a definitive large-scale trial on patients with patellofemoral pain, within the NHS in the UK. We will identify strengths and weaknesses of the proposed protocol and the utility and characteristics of the outcome measures. The results from this study will inform the design of a multicentre trial.

**Trial registration:**

ISRCTN35272486.

## Background

Patellofemoral pain (PFP) is one of the most common forms of knee pain in adults under the age of 40 [1–3], with an estimated prevalence of 23% in the general population [1]. Typically, symptoms include retropatella pain, or diffuse peripatellar pain, aggravated by activities that load the joint, such as climbing and descending stairs, squatting and running [4].

The overall long-term prognosis for the majority of patients with PFP is poor [5]. One third of patients are pain-free 1 year after the diagnosis [5]. Patients will typically withdraw from participation in sport and leisure activities [6, 7], and symptoms can continue for many years [5, 8]. Furthermore, many individuals with PFP develop associated psychological distress, such as fear avoidance and catastrophising thoughts in relation to their knee pain [9–11]. It is such a common, yet poorly understood condition, that the Chartered Society of Physiotherapy, UK (CSP), has ranked PFP the 3rd most important topic out of 185 in their Musculoskeletal Research Priority Project in 2012 [12].

There remains scientific debate and uncertainty around the underlying aetiology of the condition [13], and it is thought most likely to be multifactorial in its origin [14]. There is currently little high-quality level 1 evidence on which to base conservative management [15]. Historically, models of clinical reasoning based on the patho-anatomical basis of tissue pathology and differential diagnosis have labelled one major cause of PFP as patellar mal-tracking/malalignment [14, 16–18], with the supposition that various tissue structures could be contributing, such as general muscle weakness [19], soft tissue tightness [20], lower limb structural abnormalities [21], movement dysfunction [22] and quadriceps mal-timing [23]. It is thought that these deviations from the ‘normal’ affect patellar alignment, kinematics or joint loading, resulting in greater stress between the patella and femur and the development of pain and dysfunction [14, 16–18]. This biomedical model of pain establishes a direct relationship between tissue structure and pain [24], and traditionally, the focus of physiotherapy treatment has been aimed at reducing pain and improving function by addressing these biomedical tissue structures; treatments including taping, stretches, exercises, electrotherapy, joint mobilisations and foot orthoses have all been suggested [16, 17]. However, systematic reviews consistently acknowledge the limitations of included studies when drawing their conclusions on the effectiveness of these interventions [14, 15, 17, 25–27]. Even in relation to exercise therapy, which has the strongest evidence-base [15], there remains insufficient evidence on which to determine the best form and dose of exercise [25].

Exercise therapy designed to load and temporarily aggravate patients’ symptoms has been shown to be beneficial for tendon pain [28], shoulder pain [29–31], low back pain [32, 33] and plantar heel pain [34]. In agreement with Nijs et al. [35], Littlewood et al. [36] hypothesised that the positive response to the painful loaded exercise programme could be attributed to the therapeutic impact upon the central nervous system. Specifically, the exercise prescribed is aimed at addressing fear avoidance and catastrophising beliefs within a framework of ‘hurt not equalling harm’, with pain rationalised as ‘de-conditioned’ tissue, thus in time, reducing the overall sensitivity of the central nervous system, with a modified pain output.

Exercise interventions for PFP have shown a ‘dose response’; characteristically, the more exercise the patient does the better their pain and functional improvement in the long term. A study in Norway (*n* = 42) looked at a high-dose regime versus a low-dose regime and concluded that there was significant benefit in the high-dose group over low dose in terms of pain and function at 12 weeks [37]. Strikingly, the 1-year follow-up showed that the high-dose group had continued to improve in terms of pain and function, while the low-dose group had deteriorated [37, 38]. This finding is supported by a more recent study looking at supervised exercises and education versus education alone [39]. In this study, 121 adolescents were randomised into the two groups, with exercise adherence monitored through attendance and weekly text messages. They demonstrated that successful outcome (defined as ‘completely recovered or strongly improved’ on a seven-point Likert scale) was directly correlated to the amount of exercise a patient did; if they completed the exercises 0–1× a week, 21% recovered compared with 55% who completed the exercises three or more times a week. A recent systematic review and meta-analysis of painful exercises versus pain-free exercises for chronic MSK pain found that protocols using painful exercises offered a small, but significant benefit over pain-free exercises in the short term and that protocols using painful exercises typically have higher loads and dose of exercise [40]. The optimal dose of exercise for the greatest improvements in PFP is still unknown [25] and warrants further investigation in relation to load and resistance.

High-quality research on exercise prescription in relation to pain mechanisms and dose response (or response to load/resistance) clearly warrants further investigation, particularly when considering the current paucity of high-quality evidence on which to determine the best form of exercise intervention for PFP.

### Purpose

The primary aim is to establish the feasibility and acceptability of conducting a definitive RCT which will evaluate the clinical and cost-effectiveness of a loaded self-managed exercise programme for people with patellofemoral pain compared to usual physiotherapy.

Secondary aims include establishing: if the devised loaded self-managed exercise programme can be delivered as planned in an NHS physiotherapy outpatient clinic; if the outcome measures are feasible to use within an NHS setting; if reliable data can be collected; a sample size calculation for an RCT; if the intervention is acceptable and tolerable to participants and physiotherapists; if it is feasible to recruit and randomise participants; the potential barriers to recruitment and the training package delivered to physiotherapists.

## Methods

### Study design

This is a single-centred, multiphase, sequential, mixed-methods trial. It incorporates an initial qualitative component, followed by a feasibility randomised controlled trial (RCT), with a final qualitative component. Reporting of this protocol will follow the SPIRIT statement [41].

Phase 1 will recruit eight to ten participants, and individual interviews will be performed with the purpose of understanding the impact of PFP with their lives. Their physiotherapy will continue as normal.

Phase 2: A clinical trial will then be conducted with 60 further participants (recruited separately to phase 1). These participants will be randomised to the intervention group or to the control group.

Phase 3: A sub group of participants (eight to ten) from phase 2, along with a sub-group of the physiotherapists involved in phase 2 (8–10), will be asked to take part in individual interviews that will explore the acceptability and feasibility of study design parameters and the intervention from phase 2.

### Recruitment

Selection of trial participants for phase 1 and phase 2 will follow the same procedure. Potential trial participants will be identified and triaged from the NHS physiotherapy waiting list at Derby Teaching Hospitals NHS Foundation Trust by the usual department physiotherapists who perform referral triage. Patients are referred from general practitioners and from orthopaedics and rheumatology hospital departments. An introductory letter accompanied by an information sheet and consent form will be sent out to potential trial participants by a member of the clinical team. This will be followed up by a telephone call from a member of the clinical team offering further information and enquiring about participation. Patients showing an initial interest will be asked questions to check if they match the inclusion criteria, and interview/assessments will be booked with the research team.

### Eligibility criteria

Based on a consensus gained from previous systematic reviews and studies [14, 39], the participants will be recruited from the waiting list according to the following criteria: men and women aged 18 to 40 who are able to give written informed consent; a clinical diagnosis of unilateral or bilateral PFP of greater than 3-month duration (if bilateral the worst knee will be investigated); and anterior or retropatellar pain reported on at least two of the following activities: prolonged sitting, ascending or descending stairs, squatting, jumping and running.

Exclusion criteria include previous knee surgery; awaiting lower limb surgery; knee ligamentous instability; history of patellar dislocation; true knee locking or giving way; reasons to suspect systemic pathology, or acute illness; pregnancy or breast feeding; patellar or iliotibial tract tendinopathy; and not able to speak or understand English.

### Interventions

#### Phase 1—interviews

Interviews will explore perceived barriers and facilitators to exercise engagement, the meaning to the participant and impact of having PFP. This may be used to tailor the intervention in phase 2 and to inform the phase 3 interviews, if appropriate.

A convenience sample of the first 10 participants recruited will be invited to discuss factors surrounding the meaning and lived experience of PFP. The interviews will occur in the participant’s home or another suitable venue of their choice. There will be an interview schedule to guide (but not lead) discussion.

#### Phase 2 —pilot clinical trial

##### Exercise intervention

The ‘experimental’ intervention is a loaded self-managed exercise programme for the knee and hip, aimed at addressing lower limb knee and hip weakness [25]. It is set within a framework of reducing fear/avoidance and with an emphasis on self-management and reducing the need for direct physiotherapy intervention.

Before any prescription of exercise, the physiotherapist will spend a period of time educating the participant about pain mechanisms. Descriptions of tissue-based pathology models of pain, e.g. patellar mal-tracking, or limb mal-alignment, will be actively discouraged and challenged by the physiotherapist. The participant will gain an evidence-based understanding of dysfunctional central nociceptive processing as an explanation of chronic and persistent pain [42] and the role and impact of fear [43]. This period of intense learning is designed to facilitate the reconceptualisation of pain, with an emphasis on descriptions of pain neuroscience rather than psychology [44], and from the perspective and context of the participant and their pain [45]. The education regarding pain mechanisms will take up a large portion of clinical time, such as to address any beliefs or fear within the participant that pain is a sign of tissue damage, and will be delivered in a Socratic teaching style [46]. It is expected that the education period will be completed in the first session, which typically lasts 30–40 min within the NHS, but participants that require further re-assurance may continue into their second session.

The exercise will be prescribed by the physiotherapist and will typically involve body weight resistance in the form of a modification of the ‘Step Down’ function test [47], a single-leg squatting exercise sideways on a step. By performing sideways, the participant will be able to use the guide of the wall and/or banister more easily, as guided by our patient and public involvement feedback. The exercise requires balance, knee extension strength, eccentric control and isometric hip strength. The participant will be advised to exercise to the point of fatigue, such that it reproduces their pain and discomfort, but ensuring the pain is manageable [48–50].

Exercise progression is guided by symptomatic response, such that the participant is advised that on cessation of the exercise, the pain should remain no worse than pre-exercise [48]. Participants with more severe pain will start on a lighter regime, and this will be guided by the baseline functional assessment by the treating physiotherapist. Participants will be advised to exercise at a level they find acceptable and tolerable. Participants are able to start exercising, if they wish, at a very low level, with little or no short-term pain increase, and progress when they feel comfortable and confident. Regression will be in the form of reduced repetitions, or lightening the exercise, for example to double leg squats 0–30° knee flexion. Progression can be in the form of increased repetitions or increasing the load by moving to plyometric exercises, such as jumping and hopping, for participants with higher sporting requirements. The physiotherapist will plan the exercise, motivate and review participant’s physical performance and expectations [35].

A single exercise approach will be used for this intervention. Poor levels of exercise adherence are well documented [51], and it has been suggested that a single exercise represents a pragmatic time saving approach [52]. Additionally, as previously discussed, the optimal dosage of an exercise prescription is unknown, and a single exercise approach may allow better monitoring of dosage and adherence. Importantly, it will enable the participant to observe others (the physiotherapist) perform the task successfully, and facilitate the development of mastery of the task. This combined with specific verbal and social persuasion from the physiotherapist, will further promote reconceptualisation of the pain, specific to the participant and their context; all thought to be key modifiers of perceived self-efficacy [53].

The participants will be advised that the exercise should be performed twice a day. The participant will be encouraged to self-direct in progressing/regressing the repetitions, as guided by their pain response, thus further internalising the locus of control and moving towards self-management [53].

Goal setting will be a central part of the intervention. The reconceptualisation of pain through the exercise intervention leads to the reconceptualization of pain in the participant’s daily activities, including sport and leisure activity, and setting goals helps this transition.

Other self-management strategies employed will be the discussion about managing ‘flare ups’ and potential or perceived barriers to successful outcomes [35, 52]. This will be through a thorough questioning and discussion with the physiotherapist and participant, questions such as the following: Is this safe for your knee? Is exercise good for you? Are you confident of completing this exercise? What do you think will happen? Why do you think that? It is thought that a discussion based on this approach will reveal the participant’s perception of exercise, and potential barriers and fears [35].

Keeping the treatment pragmatic, timing over follow-ups, the number of treatment sessions, frequency and discharge and physiotherapy concomitant treatments will be at the discretion of the qualified physiotherapist, but with the aim of the programme being self-management, self-directed exercise and discouraging concomitant treatments. The mean number of sessions for physiotherapy treatment of PFP is eight [54], and the prediction is that self-management strategies should lower the expected number of treatment sessions for the intervention group to three to five sessions. The timings of the follow-up appointments are also pragmatic in nature and at the discretion of the physiotherapist in discussion with the participant. Following the problem solving and barrier discussion, the physiotherapist will have understanding of the participant’s ongoing perception of their pain. Those that require further re-assurance may return sooner, 1 to 3 weeks, and those that are comfortable to self-manage sooner will return after a longer period of time, 4 to 6 weeks, or not at all in some cases. All participants have the opportunity to telephone for support if required.

To avoid cross-contamination between the two groups, the delivered intervention group will be treated by different qualified physiotherapists, who will be excluded from treating participants from the comparator group. Furthermore, physiotherapists treating ‘usual physiotherapy’ will not receive the intervention training package. The intervention training package will be delivered to the treating physiotherapists by the research team. The training package will consist of 2 × 2 h sessions, scheduled to fit into the usual in-service training slots.

##### The comparator

Usual physiotherapy typically involves strengthening exercises [55–57], taping [17, 58], stretches [59] and foot orthoses [60], and these are often aimed at restoring the assumed patella malalignment [16, 17]. The comparator will be usual physiotherapy as directed by the normal assessment and clinical decision-making by the treating physiotherapist. Details about the nature of the treatments will be collected.

#### Phase 3—interviews

Potential interviewees will be purposively sampled from the phase 2 RCT with initial contact made via telephone to ask whether they would be willing to participate (information sheet and consent form for phases 2 and 3 are combined). A sample of eight to ten participants will be required with a sample of responders and non-responders, from both intervention arm and comparator group, with 1:2 proportion of males to females to reflect gendered differences in prevalence [1]. Participants lost to follow-up will be telephoned and encouraged to take part. This process will begin 6 months after randomisation. If the participant agrees, a convenient time will be arranged to complete an interview at the participant’s home or physiotherapy clinic. Consent for the phase 2 clinical trial will include consent for participation in phase 3.

Treating physiotherapists will be invited to take part in interviews. A purposive sample of eight to ten will be required, with a mixture representing both the intervention group and comparator group, with different grades and length of clinical experience. Consent for this will be taken separately.

The emphasis within the intervention arm is towards self-management and exercises that are performed with pain. Therefore, the aim of the qualitative investigation is to give an insight into the participants’ and physiotherapists’ perceptions and experiences of the process. There have been recent developments on our understanding of the impact of patients’ and therapists’ attitudes and beliefs on pain [61]; therefore, these factors are extremely important to understand. Also, study design parameters will be discussed to explore recruitment and randomisation in this participant group. All interviews will be face-to-face at a location and time convenient to the interviewee. Interviews will be semi-structured and will broadly consider the acceptability and practicality of the exercise programme. For participants, data collection will consider views on the nature and form of the exercise; perception of its benefits, difficulties and barriers; and perceptions of study design, i.e. recruitment, consent, data collection and follow-up periods. For physiotherapists, data collection will consider views on the nature and form of the exercise; perception of its benefits, difficulties and barriers; and views on the delivery of the training package. The interviews will be guided by a semi-structured schedule.

### Outcome assessments

Our patient and public involvement representative has reviewed and approved the outcome assessments, and has a total estimated completion time of 10–15 min. The schedule for assessments is found in the SPIRIT figure (Table 1).

**Table 1:**
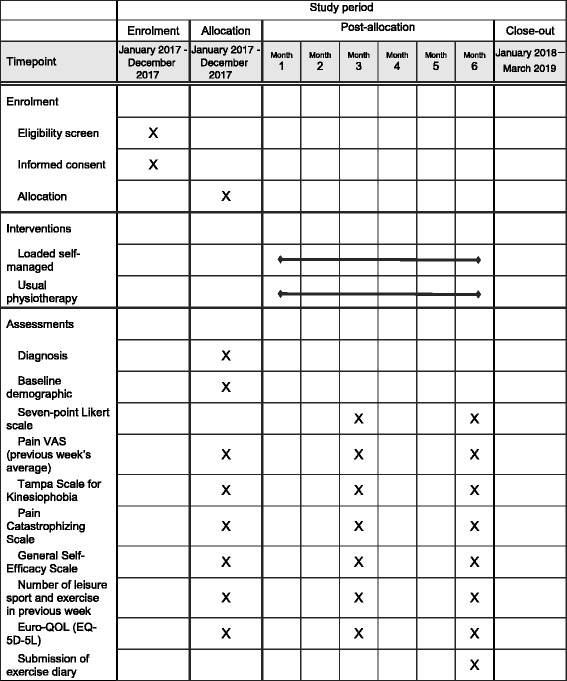
SPIRIT figure. Schedule of enrolment, interventions and assessments

Baseline demographic data will include age, sex and duration of symptoms. The primary outcome measure that we will test the feasibility of will be a global rating of change at follow-up, the proportion of participants who have recovered (defined as ‘completely recovered’ or ‘strongly recovered’), measured on a 7-point Likert scale ranging from ‘completely recovered’ to ‘worse than ever’ [39, 57, 62].

Secondary outcome measures that we will test the feasibility of using will include: visual analogue scale (VAS) for pain, kinesiophobia, catastrophizing, self-efficacy, sport participation and the generic health outcome Euro-QOL (EQ-5D-5L).

Average pain within the last week will be measured on the visual analogue scale (VAS) 0 to 10 cm [63]; 0 represents no pain, 10 the worst pain possible; this scale has been shown to be valid for PFP with a minimal clinically important difference (MCID) of 2 [64].

Fear associated with avoidance behaviours and kinesiophobia will be measured with the Tampa Scale for Kinesiophobia (TSK) [9, 65]. This is a 17-item questionnaire widely used for the assessment of fear of movement and has been shown to be reliable and valid for an English speaking population with spinal pain. Each question is scored on a 4-point Likert scale ranging from ‘strongly disagree’ (1) to strongly agree (4), giving a total possible score of 17 to 68.

Catastrophizing will be measured by means of the ‘Pain Catastrophizing Scale’ (PCS) [66]. The PCS scale is a 13-item questionnaire used to explore participants’ thoughts and feelings when experiencing pain. Each question asks the degree with which the participant agrees with the statement and is scored on a 5-point Likert scale ranging from ‘not at all’ (0) to ‘all the time’ (4), giving a total possible score of 0 to 52.

Self-efficacy has been shown to be a strong predictor of disability in patients with MSK pain [67]; therefore, the General Self Efficacy Scale will also be used (GSES) [68]. The GSES is a 10-item questionnaire with each question asking the degree with which the participant agrees with the statement, with a 4-point Likert scoring structure ranging from ‘not at all’ (1) to ‘exactly true’ (4). The questions are used to explore the participants’ perceived belief at coping with a range of stressful and challenging demands, with a total possible score of 10 to 40. The GSES has been shown to have high reliability and validity across multiple languages and settings [69].

Patients characteristically withdraw from participation in sport and leisure activities [6]; therefore, the number of times the participant has participated in leisure time sport or exercises within a week will be recorded.

The generic EQ-5D-5L is a generic health outcome used widely internationally [70]. The questionnaire has five questions about mobility, usual activities, self-care, pain and discomfort and anxiety and depression. The results are converted into in single summary index and can be used to aid and assist any future economic evaluation planned for the definitive RCT.

Compliance is the act of conforming and following the prescribed dosage, timing and frequency of the exercise. Feasibility outcomes of compliance levels will be monitored through a participant activity diary. Participants will be asked to complete an exercise diary daily for 6 months indicating how many repetitions they completed of their exercise.

Non-responders will be telephoned after 7 days to encourage them to complete the forms and return them.

### Sample size

A formal sample size calculation will not be performed since this is a feasibility study. We therefore envisage being able to recruit 30 participants into each treatment arm, and we consider that this will give a robust and useful amount of information [71]. Part of the feasibility study is to investigate the feasibility of recruitment. However, we envisage recruiting 60 participants in 13 months.

We will use the primary outcome measure, the global rating of change scale, to inform a sample size calculation for a definitive RCT.

### Randomisation

Patients who fulfil the inclusion and exclusion criteria, read and understood the patient information sheet and have given written consent to take part in the trial will be randomised to either the intervention or the control. A web-based randomisation service with secure password protected login using random variable block-size will be used.

Due to the nature of therapeutic studies, blinding of the participants and physiotherapists is not possible [72].

### Data collection methods

#### Phases 1 and 3

Interviews will be recorded with a digital recorder. The interviews will then be transcribed verbatim and analysed.

#### Phase 2

Baseline data will be captured prior to randomisation in the physiotherapy clinic. Follow-up assessments will be 3 and 6 months (by post with a stamped addressed envelope for return). Participants will be asked to post back their exercises diary at the 6-month follow-up.

### Planned data analysis

#### Phases 1 and 3

The qualitative components will follow a thematic analysis approach, as described by Braun and Clarke (2006) [73]. Line by line coding, leading to a thematic analysis (using an abductive research strategy), will be used. Following data familiarisation, initial codes will be generated and peer reviewed, by a member of the research group, to search for common themes. This will be carried out using the NVivo software (NVivo qualitative data analysis Software; QSR International Pty Ltd., Version 11, 2015). For phase 1, the analysis will assess the lived experience of PFP; for phase 3, the analysis will broadly assess acceptability and feasibility of study design, intervention and training package to physiotherapists.

#### Phase 2

The analysis will focus on descriptive statistics and confidence intervals for the variables we are obtaining. The characteristics of the participants will also be described using means, standard deviations and ranges for quantitative variables and counts and proportions for categorical variables.

As this is a feasibility study, we are testing our ability to collect data; therefore, no data imputation will be performed to account for any missing data.

Feasibility threshold will be set at 75% for recruitment. Feasibility threshold will be set at 75% to assess reliability and completeness of outcome measures. Data relating to timing of the return of outcome forms, department referral rate, recruitment rate and numbers lost to follow-up will be recorded. Acceptability and tolerability of the treatment intervention will be assessed through completeness of outcome measures and feedback from the phase 3 qualitative interviews.

### Monitoring

The exercise intervention is low risk and is commonly used in the population. The occurrence of an adverse event as a result of participation within this study is not expected, and no adverse event data will be collected.

### Patient and public involvement

This research project has been driven by the views of people suffering from patellofemoral pain (PFP). Patients who receive physiotherapy for PFP have been consulted for their views, including patient members of the Steering Group Committee. Thoughts and preferences to current programmes of therapy and treatment have been requested, and these views have been incorporated into the planning, design, application and dissemination of this study.

Patients stressed the importance of ensuring minimal number of exercises for improved adherence; the tailoring of the physiotherapy intervention around their usual sport/hobbies (where appropriate); and the capability of telephone support when required at short notice.

The main exercise of the intervention itself was adapted after consultation with patients. Initially, the intervention was an exercise based upon the ‘Step Down’ function test [47]. The feedback from the patients was that performing the same manoeuvre sideways, rather than facing down the step, allowed them to use the guide of the wall and/or banister with their hands. This has been incorporated into the intervention.

## Discussion

We have presented the rationale and design of a mixed-methods feasibility study for a loaded self-managed exercise programme for PFP. The premise that a loaded self-managed lower limb strengthening exercise, that is not directed at specific tissue pathology, but rather based on the neurophysiology of pain, set within clearly defined boundaries, will have a positive impact upon fear-avoidance, catastrophizing and self-efficacy behaviour and patient-reported pain levels. The feasibility of a large definitive RCT will either be established or negated, and the results of the trial will be published when they are available.

## Abbreviations

CKRS: Cincinnati knee rating system
CSP: Chartered Society of Physiotherapy, UK
EQ-5D-5L: Euro-QOL
GSES: General self-efficacy scale
HEE: Health Education England
MCID: Minimal clinically important difference
MRI: Magnetic Resonance Imaging
MSK: Musculoskeletal
NHS: National Health Service
NIHR: National Institute for Health Research
PCS: Pain catastrophizing scale
PFP: Patellofemoral pain
RCT: Randomised controlled trial
TSK: Tampa Scale for Kinesiophobia
VAS: Visual analogue scale
